# Whole brain radiation therapy does not improve the overall survival of EGFR-mutant NSCLC patients with leptomeningeal metastasis

**DOI:** 10.1186/s13014-019-1376-z

**Published:** 2019-09-14

**Authors:** Weiwei Yan, Yang Liu, Ji Li, Anqin Han, Li Kong, Jinming Yu, Hui Zhu

**Affiliations:** 1 School of Medicine and Life Sciences, University of Jinan-Shandong Academy of Medical Sciences, Jinan, Shandong China; 2 Department of Radiation Oncology, Shandong Cancer Hospital and Institute, Shandong First Medical University and Shandong Academy of Medical Science, Jinan, Shandong China

**Keywords:** Non-small cell lung cancer, Leptomeningeal metastasis, EGFR mutations, WBRT, Survival, Treatment response

## Abstract

**Background:**

Leptomeningeal metastasis (LM) is a devastating and terminal complication of advanced non-small-cell lung cancer (NSCLC), especially in patients harboring epidermal growth factor receptor (EGFR) mutations. The role of whole brain radiation therapy (WBRT) in the treatment of EGFR-mutant NSCLC patients with LM is not conclusive. Therefore, we conducted a retrospective study to evaluate the therapeutic effect of WBRT in this setting.

**Methods:**

EGFR-mutant NSCLC patients with LM, who had previously received treatment at the Shandong Cancer Hospital and Institute from July 2014 to March 2018 were reviewed retrospectively. LM was diagnosed by positive CSF cytology and/or leptomeningeal-enhanced magnetic resonance imaging (MRI). Survival was estimated using the Kaplan-Meier method.

**Results:**

In total, 51 EGFR-mutated NSCLC patients with LM were eligible for analysis, subdivided into 26 in the WBRT group and 25 in the non-WBRT group. No significant differences were observed in intracranial ORR (15.4% vs. 16%, *p* = 0.952) and DCR (34.7% vs. 28%, *p* = 0.611) between the two groups. The median iPFS_LM_ and OS_LM_ for the entire cohort were 3.3 months (95% CI: 2.77–3.83) and 12.6 months (95% CI: 9.66–15.54), respectively. No difference in iPFS_LM_ was observed between the WBRT and non-WBRT groups (median 3.9 vs. 2.8 months; HR = 0.506, *p* = 0.052). The median OS_LM_ was 13.6 months in the WBRT group, compared with 5.7 months in the non-WBRT group (HR = 0.454, *p* = 0.022). Multivariate analyses of OS_LM_ showed that KPS ≥ 80 at the time of LM diagnosis (HR = 0.428, 95% CI: 0.19–0.94; *p* = 0.034) and the administration of EGFR-TKIs (HR = 0.258, 95% CI: 0.11–0.58; *p* = 0.001) were independent predictors of survival, but WBRT (HR = 0.49, 95% CI: 0.24–1.01; *p* = 0.54) was not. Toxicities associated with WBRT or other treatment were rare.

**Conclusion:**

For EGFR-mutated NSCLC patients with LM, WBRT did not improve intracranial treatment response and survival statistically.

## Introduction

Leptomeningeal metastasis (LM), also termed as neoplastic meningitis, is caused by the diffusion of malignant cells to the leptomeninges and the cerebrospinal fluid (CSF) [1–4]. Approximately 3.8% of patients with advanced NSCLC have LM at the date of diagnosis or in the course of disease. The incidence of LM in patients harboring epidermal growth factor receptor (EGFR) mutations (9.4%) is higher than that in patients with wild-type EGFR (1.7%) [5, 6]. LM often represents a terminal event of NSCLC associated with an extremely poor prognosis, and the median OS of unselected NSCLC patients varies between 3 and 6 months [6–10].

Currently, the treatment for LM consists of using either EGFR-tyrosine kinase inhibitors (TKIs), whole brain radiation therapy (WBRT), Chemotherapy (ChT), intrathecal chemotherapy (ITC), and ventriculoperitoneal-shunt (VP-shunt) [5, 6, 8, 10–12]. However, the survival benefits of these treatments remain poorly established. The poor permeability of chemotherapeutic or targeted agents through the blood-brain barrier (BBB) may account for the limited role of these treatments. Although WBRT is an effective treatment for patients with multiple brain metastases (BMs), its therapeutic effect in LM patients with EGFR mutations has not been evaluated fully.

Even though most previous studies have been conducted in unselected NSCLC patients, the survival data of LM in EGFR-mutant NSCLC in those studies were in shortage. Therefore, we performed a retrospective analysis of the clinical data of EGFR-mutated NSCLC patients with LM who had received treatment at our hospital from July 2014 to March 2018, aiming to evaluate whether WBRT could provide survival benefits for LM patients with EGFR mutations.

## Patients and methods

### Patients

Medical records of EGFR-mutant NSCLC patients with cytologically or radiographically confirmed LM treated at the Shandong Cancer Hospital and Institute between July 2014 and March 2018 were collected for this investigation. All patients were diagnosed pathologically with NSCLC. The EGFR status was identified from primary lung tumors using the amplification refractory mutation system (ARMS) analysis. LM diagnosis was based on the detection of malignant cells in the CSF, the focal or diffuse enhancement of leptomeninges, and nerve roots or the ependymal surface on gadolinium-enhanced MRI. The medical ethical committee of the Shandong Cancer Hospital and Institute approved the study protocol.

### Clinical data collection

Data for patient characteristics, tumor features, treatment modalities, and survival outcomes were extracted from medical records. Depending on whether they had received WBRT, enrolled patients were divided into a WBRT group and a non-WBRT group. Patients in the two groups were categorized according to age, gender, smoking status, Karnofsky performance status (KPS) at the time of LM diagnosis, extracranial metastases, NSCLC pathological classification, EGFR mutation status, co-existing BM, and LM-related symptoms and signs at the time of LM diagnosis.

### Intracranial response evaluation

Complete medical histories were obtained, and physical examinations, laboratory examinations, and brain MRI were performed and evaluated before treatment. Lumbar puncture was recommended for the assessment of LM, but it was not mandatory. LM evaluations were performed 1 month after beginning treatment and were followed up once every 2–3 months, or at the time of neurological deterioration. LM response assessment criteria are usually based on published randomized clinical trials, including MRI outcomes, neurological symptoms, and CSF parameters (intracranial pressure; levels of protein, glucose, and chloride; qualitative and quantitative cytology) [13].

In the present study, not all patients were evaluated based on CSF parameters because frequent lumbar puncture was not feasible in some patients. We used MRI outcomes and neurological symptoms to evaluate LM clinical responses. MRI imaging evaluations show subarachnoid masses as measurable lesions and linear or diffuse meningeal enhancement as unmeasurable lesions [13]. The assessment of neurological functions covered cerebral hemisphere symptoms, cranial nerve symptoms, and spinal cord and root symptoms.

A complete response (CR) was defined as the disappearance of all meningeal lesions and neurological symptoms. A partial response (PR) was defined as the improvement in neurological symptoms and a 50% or more shrinkage in the bidirectional measurement of subarachnoid masses. A stable disease (SD) was defined as stable neurological symptoms and a subarachnoid mass shrinkage of less than 50% or an increase of less than 25%. One of the following three denoted a progressive disease (PD): a neurological progression, a subarachnoid mass increase of 25% or more, or an unequivocal progression of existing unmeasurable lesions. The objective response rate (ORR) of intracranial lesions included the combination of CR and PR, and the disease control rate (DCR) of intracranial lesions included CR, PR, and SD.

### Evaluation of treatment toxicity

The treatment toxicity associated with WBRT or other treatment were evaluated based on the CTCAE 4.0 edition (Common Terminology Criteria for Adverse Events version 4.0) every month, with toxicity graded as mild (grade 1), moderate (grade 2), severe (grade 3) or life-threatening (grade 4).

### Statistical analysis

All parameters were analyzed as dichotomous variables. Baseline characteristics of patients from the WBRT group and non-WBRT group were compared using the Chi-square or Fisher’s exact tests. Intracranial progression-free survival (iPFS_LM_) was calculated from the date of LM diagnosis to the first documentation of intracranial lesion progression or death with documented intracranial progression. Overall survival (OS_LM_) was calculated from the date of LM diagnosis to death from any cause or last follow-up. The Kaplan-Meier method was used to estimate survival, and the log-rank test was used to compare differences in survival between subgroups. Cox’s proportional hazards model was used to evaluate the independent prognostic factors associated with improved survival. All statistically significant variables in the univariate analysis were subjected to the multivariate Cox regression analysis. A *p*-value < 0.05 was considered to show statistical significance in all analyses. All statistical analyses were performed using SPSS Statistics version 20 (IBM Corporation, NY, USA).

## Results

### Clinical characteristics

Fifty-one EGFR-mutated NSCLC patients with LM were eligible for analysis, subdivided into 26 in the WBRT group and 25 in the non-WBRT group. The baseline characteristics of the two groups are summarized in Table 1. For all baseline characteristics, only EGFR mutation types had statistically different distributions between the two groups (*p* < 0.05). From the entire cohort, 9 (17.6%) patients had LM at the initial diagnosis of metastatic NSCLC, with the remaining patients were diagnosed with LM during the disease. The median time from the diagnosis of NSCLC to LM was 17.4 months (95% CI: 8.89–25.92, range 0 to 88.9 months). Twenty patients (39.2%) had the EGFR exon 19 deletion mutation, and 31 (60.8%) had the exon 21 L858R mutation. All patients showed typical MRI manifestations of LM. Forty (78.4%) patients were diagnosed with LM by MRI and CSF cytology, and 11 (21.6%) patients were diagnosed with LM by MRI alone. Forty-four (86.3%) patients were diagnosed with both LM and BM; 22 of them developed BM before LM, 21 were diagnosed with BM and LM at the same, and one was diagnosed with BM after LM.

**Table 1:** Baseline characteristics of enrolled patients

Characteristic	Total (*n* = 51) n (%)	WBRT (*n* = 26) n (%)	non-WBRT (*n* = 25) n (%)	*P*-value
Age at the time of LM diagnosis (yr.)				
Median (range)	56 (32–81)	55 (32–81)	56 (37–73)	0.843
< 60	34 (66.7)	17 (65.4)	17 (68)	
≥ 60	17 (33.3)	9 (34.6)	8 (32)	
Gender				
Male	17 (33.3)	8 (30.8)	9 (36)	0.692
Female	34 (66.7)	18 (69.2)	16 (64)	
Smoking status				
Non-smoker	40 (78.4)	21 (80.8)	19 (76)	0.679
Former/current-smoker	11 (21.6)	5 (19.2)	6 (24)	
KPS at the time of LM diagnosis				
≥ 80	39 (76.5)	21 (80.8)	18 (72)	0.46
< 80	12 (23.5)	5 (19.2)	7 (28)	
Pathological classification				
Adenocarcinoma	50 (98)	26 (100)	24 (96)	0.49
Squamous cell carcinoma	1 (2)	0 (0)	1 (4)	
EGFR mutation status				
Exon 19 deletion	20 (39.2)	5 (19.2)	15 (60)	0.004
Exon 21 L858R	31 (60.8)	21 (80.8)	10 (40)	
LM present at the initial diagnosis of NSCLC				
Yes	9 (17.6)	6 (23.1)	3 (12)	0.465
No	42 (82.4)	20 (76.9)	22 (88)	
LM-related Symptoms and signs				
Asymptomatic	12 (23.5)	4 (15.4)	8 (32)	0.199
Symptomatic	39 (76.5)	22 (84.6)	17 (68)	
The modality of LM diagnosis				
MRI+	11 (21.6)	6 (23.1)	5 (20)	0.789
MRI+/cytology+	40 (78.4)	20 (76.9)	20 (80)	
Co-existing BMs				
Yes	44 (86.3)	24 (92.3)	20 (80)	0.248
No	7 (13.7)	2 (7.7)	5 (20)	
Extracranial metastases at the time of LM diagnosis				
Yes	37 (72.5)	17 (65.4)	19 (76)	0.406
No	14 (27.5)	9 (34.6)	6 (24)	

*LM* Leptomeningeal metastasis, *KPS* Karnofsky performance status, *NSCLC* Non-small-cell lung cancer, *EGFR* Epidermal growth factor receptor, *MRI* Magnetic resonance imaging, *BMs* Brain metastases

### Treatments

Thirty Gy of WBRT was delivered to patients in the WBRT group in 10 fractions for 5 days per week. Twenty patients received EGFR-TKIs and WBRT, 3 patients underwent WBRT plus ChT, and 3 patients underwent WBRT alone. In the non-WBRT group, 9 patients received EGFR-TKIs alone, 9 patients underwent ChT alone, and 7 patients received both EGFR-TKIs and ChT (Table 2). Among 36 patients received EGFR-TKIs, 13 received gefitinib, 19 received erlotinib, and 4 received icotinib.

**Table 2:** Treatment methods for the enrolled patients

	WBRT (*n* = 26)	non-WBRT (*n* = 25)
WBRT alone	3	0
EGFR-TKIs alone	0	9
ChT alone	0	9
WBRT+EGFR-TKIs	20	0
WBRT+ChT	3	0
EGFR-TKIs+ChT	0	7

*EGFR* Epidermal growth factor receptor, *TKI* Tyrosine kinase inhibitor, *ChT* Chemotherapy, *WBRT* Whole brain radiotherapy

### Intracranial treatment response

The intracranial treatment responses in the two groups are summarized in Table 3. All patients were evaluated radiologically and clinically after treatment for LM. In the WBRT group, 2 patients (7.69%) had CR, 2 (7.69%) had PR, 5 (19.23%) had SD, and 17 (65.39%) had PD, whereas, it was 1 (4%), 3 (12%), 3 (12%), and 18 (72%) in the non-WBRT group for CR, PR, SD, and PD, respectively. Intracranial ORRs were similar between the two groups (15.4% vs. 16%, *p* = 0.952). Intracranial DCR was 34.7% for the WBRT group and 28% for the non-WBRT group (*p* = 0.611). No significant differences were observed in intracranial ORR and DCR between the two groups.

**Table 3:** Intracranial Treatment Response for the enrolled patients

	All patients (*n* = 51)	WBRT (*n* = 26)	non-WBRT (*n* = 25)	*P*-value
CR	3 (5.88%)	2 (7.69%)	1 (4%)	
PR	5 (9.8%)	2 (7.69%)	3 (12%)	
SD	8 (15.69%)	5 (19.23%)	3 (12%)	
PD	35 (68.63%)	17 (65.39%)	18 (72%)	
ORR	8 (15.7%)	4 (15.4%)	4 (16%)	0.952
DCR	16 (31.4%)	9 (34.7%)	7 (28%)	0.611

*CR* Complete response, *PR* Partial response, *SD* Stable disease, *PD* Progression disease, *ORR* Objective response rate, *DCR* Disease control rate

### Toxicity and safety

In our study, all 51 patients had good tolerance to the treatment regimen. No patient suspended or interrupted treatment due to serious adverse event. The most frequent acute toxicities associated with WBRT included grade 1 headache in 6 patients, grade 1 nausea/vomiting in 8 patients, and grade 1 dermatitis in 3 patients. No late neurocognitive impairment and reduced quality of life related to WBRT has been detected. The most common adverse reactions of EGFR-TKIs treatment were grade 1 or grade 2 rash, diarrhea, and nausea. Rash occurred in 7 patients, diarrhea in 6 patients, and nausea in 3 patients. The patients had good tolerance to WBRT combined with EGFR-TKIs/ChT and EGFR-TKIs combined with ChT. No patient experienced grade ≥ 3 treatment-related toxicity in this cohort of patients.

### Intracranial progression-free survival

**Fig. 1:**
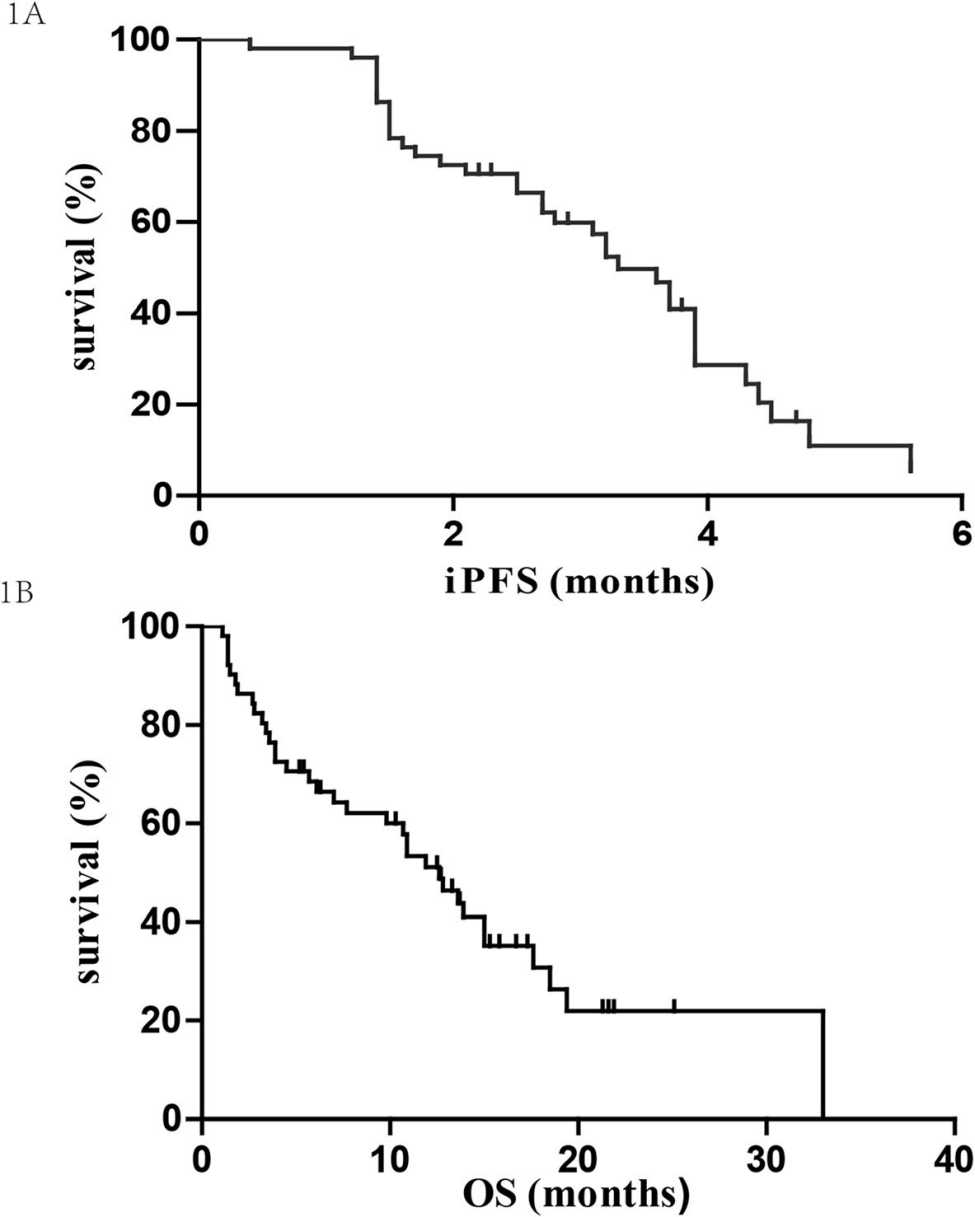
Intracranial progression-free survival (**a**) and overall survival (**b**) after the diagnosis of LM

**Fig. 2:**
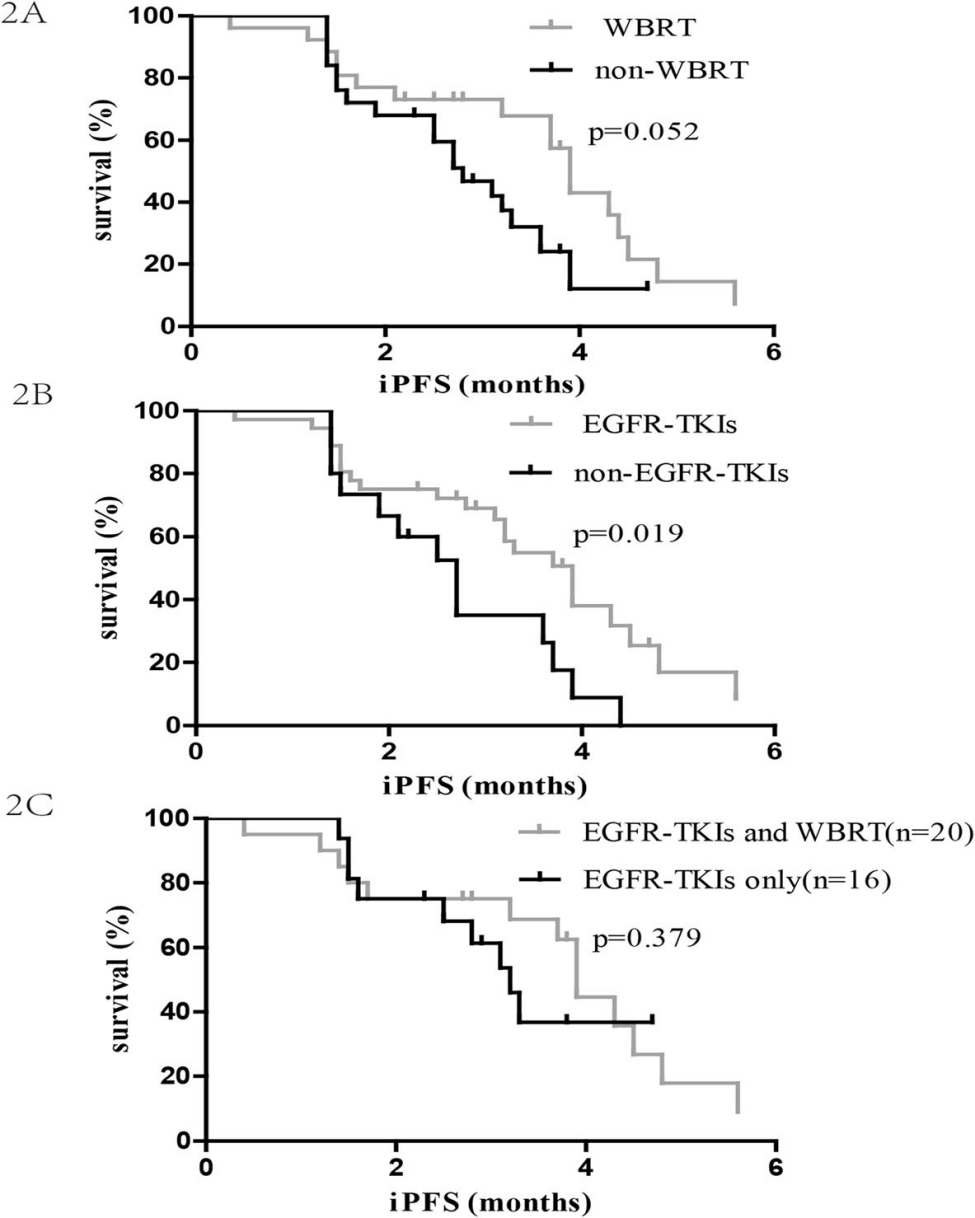
The Kaplan-Meier analysis showing the intracranial progression-free survival of all patients. **a** survival of patients who received WBRT compared with those who did not; **b** survival of patients who received EGFR-TKIs compared with those who did not; **c** survival of patients who received WBRT plus EGFR-TKIs compared with those who received EGFR-TKIs alone

The median iPFS_LM_ for the entire cohort was 3.3 months (95% CI: 2.77–3.83; Fig. 1a). Intracranial progression was detected in 68.63% (25 of 51) patients; 65.39% (17 of 26) had intracranial progression in the WBRT group, while it was 72% (18 of 25) in the non-WBRT group. The difference in iPFS_LM_ between the two groups was not statistically significant (median 3.9 vs. 2.8 months; *p* = 0.052; Fig. 2a). The 36 patients who received EGFR-TKIs demonstrated longer iPFS_LM_ than those without EGFR-TKIs (median 3.9 vs. 2.7 months; *p* = 0.019; Fig. 2b). The median iPFS_LM_ of patients who underwent EGFR-TKIs and WBRT was similar to that of patients who underwent only EGFR-TKIs (median 3.9 vs. 3.2 months; *p* = 0.379; Fig. 2c).

In multivariate analyses, KPS ≥ 80 at the time of LM diagnosis (HR = 0.338, 95% CI: 0.15–0.78; *p* = 0.011) and the administration of the EGFR-TKI therapy (HR = 0.442, 95% CI: 0.22–0.91; *p* = 0.027) were independent predictors associated with favorable survival. Additional results are detailed in Table 4.

**Table 4:** Univariate and multivariate analyses of clinical variables on intracranial progression -free survival

	Univariate HR (95% CI)	*P*	Multivariate HR (95% CI)	*P*
Age (≥60/< 60)	1.66 (0.78–3.54)	0.17		
Gender (Female/Male)	0.64 (0.31–1.31)	0.204		
Smoking status (Smoking/Never)	1.41 (0.65–3.06)	0.375		
KPS (≥80/< 80)	0.34 (0.15–0.78)	0.006	0.34 (0.15–0.78)	0.011
EGFR mutation (exon 19 deletion/exon 21 L858R)	0.77 (0.39–1.52)	0.43		
Co-existing BMs (yes/no)	0.89 (0.34–2.33)	0.804		
Intracranial symptoms (yes/no)	1.40 (0.61–3.22)	0.413		
LM at the time of NSCLC diagnosis (yes/no)	0.60 (0.21–1.72)	0.324		
Treatment for LM				
WBRT (yes/no)	0.51 (0.25–1.04)	0.052		
EGFR-TKIs (yes/no)	0.45 (0.22–0.91)	0.019	0.442 (0.22–0.91)	0.027
WBRT+EGFR-TKIs/EGFR-TKIs alone	0.67 (0.27–1.67)	0.379		

*LM* Leptomeningeal metastasis, *KPS* Karnofsky performance status, *NSCLC* Non-small-cell lung cancer, *EGFR* Epidermal growth factor receptor, *TKI* Tyrosine kinase inhibitor, *WBRT* Whole brain radiotherapy, *ChT* Chemotherapy, *BMs* Brain metastases

### Overall survival

**Fig. 3:**
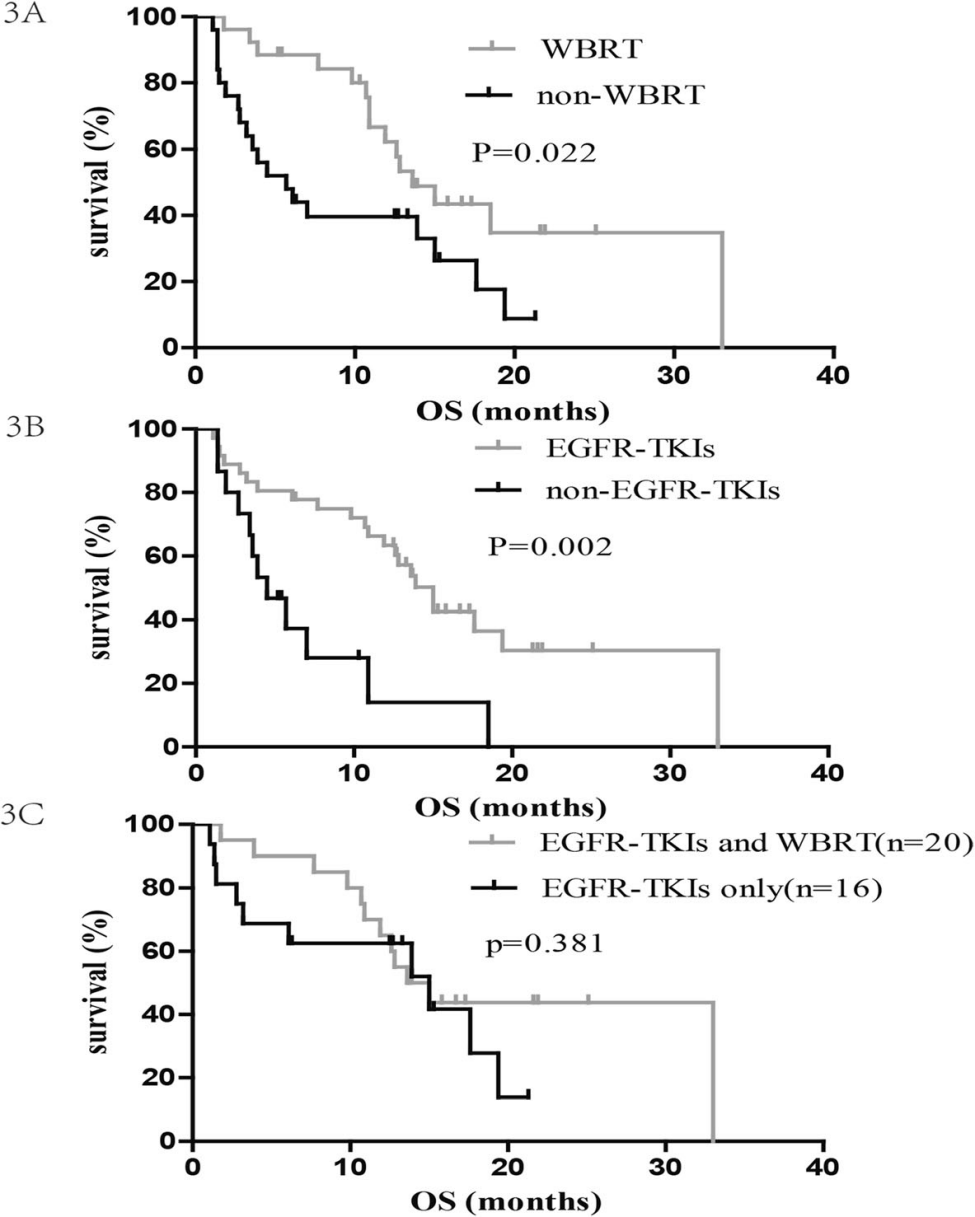
The Kaplan-Meier analysis showing the overall survival of all patients. **a** survival of patients who received WBRT compared with those who did not; **b** survival of patients who received EGFR-TKIs compared with those who did not; **c** survival of patients who received WBRT plus EGFR-TKIs compared with those who received EGFR-TKIs alone

The last follow-up date was carried out on June 30, 2018. Seventeen patients were still alive by the end of the follow-up. The median OS_LM_ for the entire cohort after LM diagnosis was 12.6 months (95% CI: 9.66–15.54; Fig. 1b). The median OS_LM_ were 13.6 and 5.7 months in the WBRT and non-WBRT groups, respectively (*p* = 0.022; Fig. 3a). The median survival time of the 36 patients who received EGFR-TKIs was 15 months, which was significantly longer than the 4.5 months of the 15 patients who did not receive EGFR-TKIs (*p* = 0.002; Fig. 3b). Nineteen patients who received erlotinib achieved significantly longer OS_LM_ than those who underwent gefitinib (median OS_LM_ 15 vs. 6.1 months, *p* = 0.012). Further analysis showed that the combination of EGFR-TKIs and WBRT did not provide any additional survival benefit, compared to patients who received only EGFR-TKIs (median 13.6 vs. 15 months; *p* = 0.381; Fig. 3c).

Results from the variables of KPS, EGFR-TKI therapy, and WBRT subjected to multivariate analysis showed that KPS ≥ 80 at the time of LM diagnosis (HR = 0.428, 95% CI: 0.19–0.94; *p* = 0.034) and the administration of the EGFR-TKI therapy (HR = 0.258, 95% CI: 0.11–0.58; *p* = 0.001) were favorable prognostic factors of OS_LM_, whereas, the use of WBRT (HR = 0.49, 95% CI: 0.24–1.01; *p* = 0.54) was not an independent predictor (Table 5).

**Table 5:** Univariate and multivariate analyses of clinical variables on overall survival

	Univariate HR (95% CI)	*P*	Multivariate HR (95% CI)	*P*
Age (≥60/< 60)	1.17 (0.58–2.36)	0.667		
Gender (Female/Male)	0.61 (0.29–1.23)	0.158		
Smoking status (Smoking/Never)	1.61 (0.74–3.51)	0.225		
KPS (≥80/< 80)	0.39 (0.19–0.84)	0.012	0.43 (0.19–0.94)	0.034
EGFR mutation (exon 19 deletion/exon 21 L858R)	0.79 (0.39–1.58)	0.495		
Co-existing BMs (yes/no)	0.57 (0.22–1.51)	0.25		
Intracranial symptoms (yes/no)	1.31 (0.54–3.17)	0.554		
LM at the time of NSCLC diagnosis (yes/no)	0.35 (0.11–1.14)	0.066		
Treatment for LM				
WBRT (yes/no)	0.45 (0.23–0.91)	0.022	0.49 (0.24–1.01)	0.54
EGFR-TKIs (yes/no)	0.31 (0.15–0.67)	0.002	0.26 (0.11–0.58)	0.001
WBRT+EGFR-TKIs/EGFR-TKIs alone	0.68 (0.29–1.61)	0.381		

*LM* Leptomeningeal metastasis, *KPS* Karnofsky performance status, *NSCLC* Non-small-cell lung cancer, *EGFR* Epidermal growth factor receptor, *TKI* Tyrosine kinase inhibitor, *WBRT* Whole brain radiotherapy, *ChT* Chemotherapy, *BMs* Brain metastases

## Discussion

This retrospective study is a relatively large cohort of EGFR-mutant LM patients focusing on the role of WBRT in the treatment of LM. The highlight of the study is the homogeneity of all LM patients harboring EGFR mutations. Our findings showed that the administration of EGFR-TKIs and KPS ≥ 80 at the time of LM diagnosis were associated with prolonged survival. However, WBRT did not improve intracranial treatment response and survival statistically for patients selected for this study.

The survival benefit and treatment response of WBRT to NSCLC patients with LM remain debatable, especially in patients with EGFR mutations [1, 5, 6, 12, 14–17]. A previous retrospective assessment of 52 EGFR-mutant LM patients showed that WBRT did not provide survival benefit [6]. In another retrospective cohort of 109 LM patients harboring EGFR mutations, patients who underwent WBRT for LM treatment did not achieve longer survival than those without WBRT (9.3 vs. 8.1 months; *p* = 0.448) [5]. Although Kuiper et al. [17] showed that WBRT could play a role in symptom control, they did not find that it influenced the survival of LM patients harboring EGFR mutations. So far, few retrospective studies have shown that WBRT could bring survival benefits to LM patients with EGFR mutations. Similarly, the survival benefit of WBRT to brain metastases is controversial. A randomized clinical trial QUARTZ showed that except for younger patients, WBRT had no significant effect on survival or quality of life in patients with brain metastases [18].

In LM, a neuraxis disease, CSF circulates dynamically through the whole compartment of the central nervous system (i.e., the intracranial and intraspinal compartments), and malignant cell diffusion affects all CSF compartments [19]. Based on the characteristics of LM, the whole craniospinal axis should be defined as target volume of radiotherapy. However, craniospinal irradiation (CSI) is rarely recommended in clinical work, because of its obvious myelotoxicity and the lack of evidence of its survival benefits [3, 20]. Subsequently, only the intracranial CSF compartment of the CNS has been irradiated in the treatment of LM with WBRT [17, 19, 21], which could account for the limited role of WBRT.

LM is more common in NSCLC patients harboring EGFR mutations, especially in patients after effective EGFR-TKI treatment [5, 9]. In our study, 23 of 30 developed LM during treatment with EGFR-TKI. After the diagnosis of LM, the 36 patients who received EGFR-TKIs achieved significantly longer iPFS_LM_ and OS_LM_ than patients who did not (median iPFS_LM_ 3.9 vs. 2.7 months, *p* = 0.019; median OS_LM_ 10 vs. 3.3 months, *p* = 0.002), and these findings are consistent with those from previous studies [5, 6, 22, 23]. Li et al. [5] showed that patients who received EGFR-TKIs after LM diagnosis had significantly longer survival compared with those who did not (10.0 vs. 3.3 months; *p* < 0.001). Another retrospective study [6] also reported that EGFR-TKI therapy was an independent predictor of longer survival in 75 EGFR-mutated NSCLC patients with LM. Overall, EGFR-TKIs exhibited good efficacy for EGFR-mutant NSCLC patients with LM.

Currently, there is no sufficient evidence to suggest that the combination of EGFR-TKIs and WBRT has a better survival benefit than EGFR-TKIs alone in EGFR-mutant NSCLC patients with LM. However, research on brain metastasis has shown that EGFR-TKIs plus WBRT has a higher response rate and significant improvement in survival, compared with EGFR-TKIs alone in the treatment of BM from EGFR-mutant NSCLC patients [24–27]. Borghetti et al. [28] also confirmed that radiation therapy combined with TKI is a safe and well-tolerated therapy for metastatic NSCLC patients with EGFR- or ALK- mutations. In this study, the addition of WBRT to EGFR-TKIs did not lead to any survival benefit in EGFR-mutant LM patients when compared with patients who received only EGFR-TKIs (median OS_LM_ 13.6 vs. 15 months; *p* = 0.381). This result is similar to the findings from 33 patients treated with both WBRT and EGFR-TKIs in a previous investigation who did not survive longer than those who received only EGFR-TKIs (median 9.7 vs. 10.1 months; *p* = 0.778, 3). To patients with EGFR-mutant LM, EGFR-TKIs alone could be the better treatment option, with further clinical studies required to certify the hypothesis.

Different EGFR-TKIs options have provided promising outcomes in the treatment of LM form NSCLC. Erlotinib reportedly could be more effective than gefitinib in the treatment of LM in NSCLC patients [29]. Compared to gefitinib, erlotinib has shown higher CSF concentrations (28.7 vs. 3.7 ng/mL, *p* = 0.0008) and penetration rates (2.77 vs. 1.13%, *p* < 0.0001) [30]. Afatinib and icotinib have also shown efficacy on LM from NSCLC with EGFR mutation [31, 32]. Osimertinib (AZD9291) is a third-generation EGFR-TKI targeting sensitized EGFR mutations and acquired EGFR T790 M resistance mutations [33]. The therapy exhibited a better BBB penetration than the other EGFR-TKIs (gefitinib, rociletinib, or afatinib) [34]. The preliminary results from the phase I BLOOM study (NCT02228369) demonstrated that high-dose osimertinib (160 mg daily) showed encouraging activity and manageable tolerability in pretreated EGFR-mutant NSCLC patients with LM confirmed by CSF cytology [35]. In another study, 20 EGFR-mutant LM patients treated with osimertinib had an OS of 18.0 months [36], whereas, OS was only 3.1 months in a cohort of 32 LM patients treated with first- and second-generation EGFR-TKIs [16]. Osimertinib appears to be more effective than the first- and second-generation EGFR-TKIs. However, the use of osimertinib in the treatment of EGFR-mutant LM has not been approved yet, and further randomized clinical studies are needed to confirm its prowess.

This study, despite its mixed findings, also has several limitations. Firstly, the retrospective design and small sample size affected its statistical power. Moreover, the data were obtained retrospectively from medical records, and there may have been biasing in patient selection. Secondly, the intracranial therapeutic response was not evaluated using CSF parameters. Therefore, our conclusions should be interpreted cautiously.

## Conclusion

In conclusion, our study revealed that EGFR-TKIs seems to have an excellent control of LM in NSCLC patients with EGFR mutations even if WBRT is not delivered; on the other hand, WBRT seems to positively influence the survival of these patients even if it does not get the statistical significance. Probably, WBRT could be omitted in asymptomatic patients when EGFR-TKIs are available for LM, thus further avoiding the side effects of WBRT.

## Data Availability

All data and materials can be found in “Patient, materials and methods” section within the article.

## Abbreviations

ARMS: Amplification refractory mutation system
BBB: Blood-brain barrier
BMs: Brain metastases
ChT: Chemotherapy
CR: Complete response
CSF: Cerebrospinal fluid
DCR: Disease control rate
EGFR: Epidermal growth factor receptor
iPFS: Intracranial progression-free survival
ITC: Intrathecal chemotherapy
KPS: Karnofsky performance status
LM: Leptomeningeal metastasis
MRI: Magnetic Resonance Imaging
NSCLC: Non-small cell lung cancer
ORR: Objective response rate
OS: Overall survival
PD: Progressive disease
PR: Partial response
SD: Stable disease
TKI: Tyrosine kinase inhibitor
VP-shunt: Ventriculoperitoneal-shunt
WBRT: Whole brain radiation therapy
